# A mediation analysis of the effect of practical training on the relationship between demographic factors, and bystanders’ self-efficacy in CPR performance

**DOI:** 10.1371/journal.pone.0215432

**Published:** 2019-04-29

**Authors:** Wonjeong Yoon, Young Sun Ro, Sung-il Cho

**Affiliations:** 1 Department of Public Health Science, Graduate School of Public Health, Seoul National University, Seoul, Republic of Korea; 2 Laboratory of Emergency Medical Services, Seoul National University Hospital Biomedical Research Institute, Seoul, Republic of Korea; 3 Department of Public Health Science, Graduate School of Public Health and Institute of Health and Environment, Seoul National University, Seoul, Republic of Korea; University of South Florida, UNITED STATES

## Abstract

This study examined the mediation effect of practical training on the relationship of demographic characteristics with bystander self-efficacy in cardiopulmonary resuscitation (CPR) performance. We used nationwide, cross-sectional data from the Korea Community Health Survey and analyzed 25,082 Korean adults who participated in CPR training within the last 2 years. A mediation model was applied to explore the pathway from demographic characteristics via CPR practical training to self-efficacy in CPR performance. A multiple logistic regression analysis was performed to examine each path in the mediation model. Of the 25,082 respondents recently trained, 19,168 (76.8%) practiced on a manikin. In the unadjusted CPR practical training model, the demographic characteristics associated with high self-efficacy in CPR performance were male gender (odds ratio [OR] = 2.54); 50s age group (OR = 1.30); college or more (OR = 1.39) and high school education (OR = 1.32); white collar (OR = 1.24) and soldier (OR = 2.98) occupational statuses. The characteristics associated with low self-efficacy were 30s age group (OR = 0.69) and capital (OR = 0.79) and metropolitan (OR = 0.84) areas of residence (p < 0.05). In the adjusted CPR practical training model, the significance of the relationship between demographics and self-efficacy in CPR performance decreased in male gender, 30s age group, college or more and high school education, and soldier occupational status (i.e., partial mediation), and disappeared in metropolitan residents (i.e., complete mediation). The degree of the mediating effect of CPR practical training on self-efficacy differed for each demographic characteristic. Thus, individualized educational strategies considering recipient demographics are needed for effective practice-based CPR training and improving bystander CPR performance.

## Introduction

Cardiopulmonary resuscitation (CPR) is a fundamental technique of basic life support and its training for the general public is emphasized by the American Heart Association guidelines [1]. CPR training is being deployed in the form of national initiatives (e.g., registration program, school curriculum, and driver’s license) in many countries, including Denmark, Sweden, Japan, and Germany, which is expected to improve bystander CPR performance and increase the survival rates from out-of-hospital cardiac arrest (OHCA) [2, 3].

Self-efficacy, a belief in one’s ability to perform CPR, is one of the indicators for evaluating the effectiveness of CPR training on actual performance [4–7]. Most previous studies aimed at improving self-efficacy have identified the relevance of CPR educational factors, such as recent training, repeated training, and practical training, and these results have been reflected in the educational policy and guidelines [1, 6, 8]. Despite the apparent effectiveness of practice-based training in cultivating self-efficacy to perform CPR in a real situation, lecture-based, mass CPR courses without the hands-on practice remain in Korea. In 2011, only 60.4% of the CPR trained Korean population had practical experience with a manikin [9]. Understanding the tendency for participation in CPR practice-based training on a demographic basis would be expected to increase the bystander CPR scale.

Independent of CPR training, another group of important factors relevant to whether a bystander can perform CPR is demographic characteristics. Previous studies identified that CPR self-efficacy performance is associated with demographic factors, such as gender, age, family status, marital status, educational level, occupation, and residential area [8–14]. The reason for these associations is that the actions taken to perform CPR involve knowledge and skills as well as personal willingness and subjective judgment [5, 10, 15]. It is necessary to consider individual characteristics and educational experience simultaneously for a better understanding of the facilitators and barriers of self-efficacy. A mediation analysis is one of the most effective methods to consider such complex relationships among three variables, and is useful for indicating how the mediator intervenes in the pathway to reach the outcome from the predictor [16]. Mediation analysis has been widely used to evaluate various educational policies and strategies, such as smoking cessation and HIV prevention, but in only a few cases has it been applied to CPR education [17–19].

In this study, we report the CPR training-mediated relationship between demographic characteristics and self-efficacy in CPR performance using a mediation analysis. This study aimed to: 1) identify demographic characteristics influencing self-efficacy in CPR performance, 2) analyze whether practical CPR training mediates particular demographic characteristics and self-efficacy of CPR performance, and 3) compare the significance and degree of the mediation effects of practical CPR training according to demographic characteristics. Finally, the results of the mediation analysis revealed the complex impact of demographic characteristics and practical CPR training experience on achieving bystander self-efficacy in CPR performance.

## Materials and methods

### Study model

**Fig 1:**
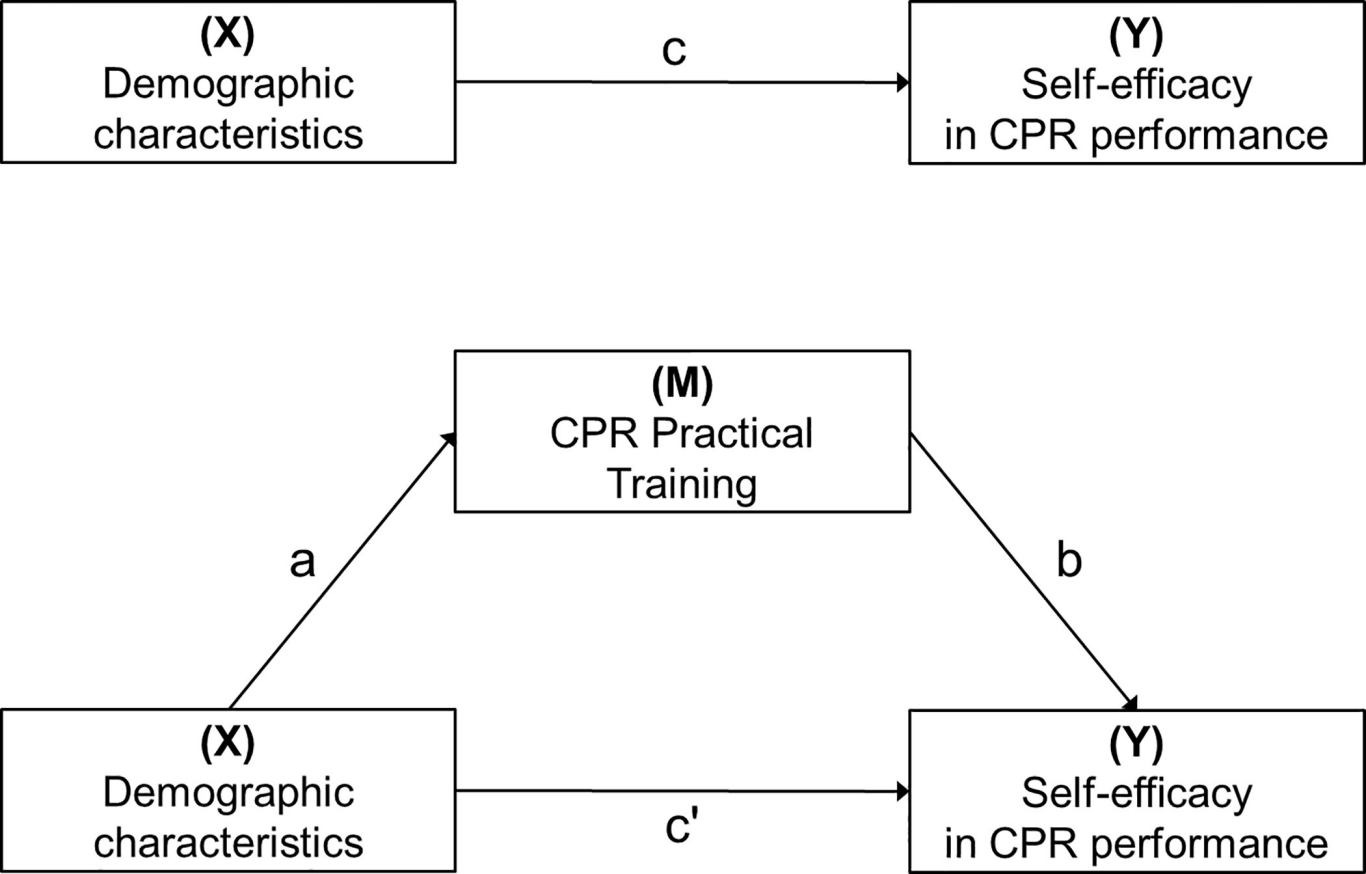
Proposed mediation model to estimate paths toward self-efficacy in CPR performance. The mediation model consisted of three paths for estimating the total effect (c), indirect effect (ab), and direct effect (c') on outcome. The demographic characteristics were gender, age group, marital status, education level, income level, occupation, and residential area.

Fig 1 shows the study model for the mediation analysis. The demographic characteristics were set as predictors (X), self-efficacy in CPR performance was the outcome (Y), and CPR practical training was the mediator (M). The traditional approach to mediation analysis consists of three regression analysis steps to reveal each relationship among three variables: 1) the first step was to find the effect (referred to as c) of X on Y (X → Y; M-unadjusted); 2) the second step was to obtain the effect (referred to as a) of X on M (X → M); and 3) the third step was to simultaneously determine the effect (referred to as b) of the X-adjusted M on Y (M → Y; X-adjusted) and the effect (referred to as c') of the M-adjusted X on Y (X → Y; M-adjusted) [20]. The effect of demographic characteristics on self-efficacy in CPR performance was called the total effect (c), and the total effect was divided into an indirect effect (ab) of demographic characteristics on self-efficacy in CPR performance through practical CPR training, and the direct effect (c') of demographic characteristics on self-efficacy in CPR performance. The mediation effect is established when the following three conditions are satisfied [20]: 1) the total effect (c) is significant; 2) the indirect effect (ab) is significant; and 3) the direct effect (c') is smaller than the total effect (c) (i.e., |c'| < |c|). In the third condition, if the direct effect is still significant, it is interpreted as ‘partially mediated,’ and if not significant, it is interpreted as ‘completely mediated’[21]. Significance was measured based on the p-value of 0.05 for each pathway [21]. However, mediation analysis with categorical variables has the problem that the value of ‘ab’ is not equal to ‘c—c'‘. To solve this problem, it is necessary to obtain a standardized coefficient, and several studies have proposed detailed processes for the calculation of this value [22–25]. By standardizing the coefficients, it is possible derive the parameters of the three-step mediation analysis that allow for the necessary comparisons.

### Study setting

The first national CPR training guidelines in Korea were published in 2006 [9]. According to these guidelines, CPR training programs for the public should include at least 40 minutes of training, including 20 minutes or more of practice using a manikin, and the maximum ratio of trainees to manikins should be two to one [26]. The rate of bystander CPR increased from 1.0% in 2006 to 6.9% in 2012, and the national OHCA survival rate increased from 2.3% in 2006 to 4.4% in 2012 [27]. However, the rate of experience with CPR education in Korea was still only 38.1% in 2011. In addition, only 60.4% of the participants had learned based on hands-on practice; the remaining 39.6% learned from theoretical training and demonstrations [6, 9, 28].

### Data

This study used data from the Korean Community Health Survey (KCHS) conducted in 2012. A total of 228,921 representative subjects were selected as participants via two-stage systematic sampling [29]. The KCHS was a nationwide, cross-sectional, and interview-type survey led by the Korea Center for Disease Control and Prevention. It has been performed annually since 2008 to investigate the health status and health behavior of adults (≥ 19 years) living in Korea. The interview was conducted by trained investigators with the aid of a computer-assisted personal interviewing system. The survey questionnaire consisted of 253 questions, which included basic personal information, socioeconomic status, residential area, disease status, and other health-related questions. The questions related to cardiac arrest and CPR were first introduced in the 2012 KCHS, and will be investigated in a 4-year cycle.

### Population

Among all KCHS respondents (N = 228,921) in 2012, those who did not respond properly to the demographic characteristics, CPR training, and self-efficacy in CPR performance items (N = 14,731) and those who did not recognize CPR itself (N = 64,746) were excluded. Of the remaining subjects (N = 149,444), data from 25,082 who had participated in CPR training within the last 2 years were analyzed to evaluate the effectiveness of practical training compared to theoretical or demonstration training.

### Variables

#### Self-efficacy in CPR performance

The question used to assess self-efficacy in CPR performance was ‘Could you perform CPR if you witnessed a cardiac arrest?’, and the possible responses were ‘I can correctly perform it’, ‘I can roughly perform it’, and ‘I cannot perform it’. Respondents who answered that they could perform CPR ‘correctly’ or ‘roughly’ were considered to have self-efficacy in CPR performance.

#### CPR practical training

The question for CPR practical training was ‘If you have been in a CPR training program for the past 2 years, have you ever practiced using a manikin in this training program?’, and the possible responses were ‘yes’ and ‘no.’ The CPR training program used here refers to a collective program of at least 40 minutes provided by accredited institutions from the CPR Association, Korea Emergency Medical Association, the Korean Red Cross, or other related organizations. The contents of the program include theoretical lectures, videos, demonstration training, and/or hands-on practice of CPR. In particular, CPR practical training means that the participants performed chest compressions on a manikin with their own hands during the course of training.

#### Demographic characteristics

The demographic characteristics used in this study included gender, age group, marital status, education level, income level, occupation, and residential area. The age groups were classified by decades: 20s (19–29 years), 30s (30–39 years), 40s (40–49 years), 50s (50–59 years), and 60s and older (≥60 years). Marital status was classified into three groups: single, married, and others (divorced, separated, and widowed). Education level was divided into college or more, high school, and less than high school. Monthly individual income level was expressed as the lowest quintile (<$599), the lower quintile ($600–1,199), the upper quintile ($1,200–1,859), and the highest quintile (≥$1,860). Occupation was divided into seven groups as white collar (professional, office, administrative), pink collar (sales, service), blue collar (technical, labor), soldier, student, housewife, and other unemployment. Residential area was classified into four major categories, including the capital (i.e., Seoul city), metropolitan, urban, and rural areas.

### Statistical analysis

We used normalized weights, stratification, and cluster variables to maintain the representativeness of our complex sample survey data [30]. The frequency analysis was performed with weighted percentages (%) to identify the distribution of subjects according to self-efficacy in CPR performance. A χ^2^ test was performed with a p-value of 0.001 to determine the relationship of self-efficacy in CPR performance with individual and educational factors. A multiple logistic regression analysis was used to explore each path in the mediation model. The results are presented as odds ratios (ORs) with 95% confidence intervals (CIs) and regression coefficient values. Here, coefficients were standardized to solve the problem of scale differences arising from the mediation analysis using the logistic regression. Standardized coefficients (β) of logistic regression models were calculated as proposed in the previous studies [24, 25], by multiplying each coefficient (B) by the standard deviation of the predictor variable and dividing by the standard deviation of the outcome variable. The standard deviation of the outcome variable is the square root of the variance of the outcome variable, which was obtained by adding the square of the pi divided by three (i.e., π^2^/3) to the variance of the predictor variable. All statistical analyses were conducted using survey analysis procedures of SAS program (version 9.4; SAS Institute, Cary, NC, USA), including SURVEYFREQ and SURVEYLOGISTIC procedures.

### Ethics

The Seoul National University Institutional Review Board approved this study (IRB no. E1801/003-003).

## Results

### Factors affecting self-efficacy in CPR performance

Table 1 shows the general characteristics of recent CPR trained subjects (N = 25,082) according to self-efficacy in CPR performance. The demographic characteristics associated with self-efficacy in CPR performance were gender, age, education level, and occupation (p < 0.001). Particularly, male; subjects in their 20s, 40s, and 50s; those with college or more and high school education; and those in white collar, blue collar, soldier, student, and unemployed occupational categories tended to have self-efficacy in CPR performance (p < 0.001). CPR practical training using manikins was related to self-efficacy in CPR performance (p < 0.001). The group receiving the practical training tended to have greater self-efficacy in CPR performance than did the group with only theoretical or demonstration training (crude OR = 4.11; 95% CI, 3.79–4.46) (S1 Table).

**Table 1:** Baseline characteristics of subjects by self-efficacy in CPR performance.

			Self-efficacy in CPR performance				
	Total		Yes		No		
	N	%w	N	%w	N	%w	p-value
Total	25082		22003		3079		
Gender							
Male	16941	73.8	15418	76.1	1523	57.7	< 0.001
Female	8141	26.2	6585	23.9	1556	42.3	
Age group (years)							
20s (19–29)	7241	37.8	6417	38.4	824	33.8	< 0.001
30s (30–39)	7632	31.8	6565	31.1	1067	36.9	
40s (40–49)	5420	17.2	4778	17.3	642	16.7	
50s (50–59)	3704	10.7	3334	10.9	370	9.6	
60s and older (≥60)	1085	2.4	909	2.3	176	3.0	
Marital status							
Single	8521	42.8	7535	43.2	986	39.9	0.001
Married	15393	53.8	13471	53.5	1922	55.9	
Others	1168	3.4	997	3.3	171	4.2	
Education level							
College or more	12137	51.7	10642	51.8	1495	51.4	<0.001
High school	10879	43.6	9607	43.8	1272	42.3	
Less than high school	2066	4.6	1754	4.4	312	6.2	
Income level							
Highest quantile	9678	42.4	8558	42.9	1120	38.9	0.003
Upper quantile	7677	30.7	6698	30.4	979	32.5	
Lower quantile	5776	20.8	5049	20.7	727	21.7	
Lowest quantile	1951	6.1	1698	6.0	253	6.9	
Occupation							
White collar	9430	40.0	8261	40.2	1169	38.9	< 0.001
Pink collar	3617	14.1	3184	14.0	433	15.2	
Blue collar	6372	21.8	5667	21.9	705	20.9	
Soldier	581	1.6	564	1.8	17	0.5	
Student	2340	12.2	2071	12.4	269	11.0	
Housewife	1549	4.9	1219	4.4	330	8.4	
Unemployed	1193	5.3	1037	5.3	156	5.0	
Residential area							
Seoul, the capital	3002	21.6	2578	21.3	424	23.4	
Metropolitan area	5222	25.0	4531	24.9	691	25.8	0.017
Urban area	8688	38.0	7618	38.2	1070	36.8	
Rural area	8170	15.3	7276	15.5	894	13.9	
CPR practical training							
Yes	19168	76.8	17625	80.5	1543	51.0	< 0.001
No	5914	23.2	4378	19.5	1536	49.0	

CPR: cardiopulmonary resuscitation

### Demographic variations in CPR practical training

Table 2 summarizes the percentage of CPR practical training using manikins according to the demographic characteristics. Of the total recently trained CPR group, 76.8% (N = 19,168) participated in practice-based CPR training. Subjects with different demographic characteristics including gender, age, education level, occupation, and residential area, were significantly different in the rate of CPR practical training (p < 0.001). Subjects in the groups that were male (OR = 1.17; 95% CI, 1.07–1.29), in their 20s (OR = 1.72; 95% CI, 1.32–2.24), with college or more (OR = 1.36; 95% CI, 1.12–1.65) or high school education (OR = 1.33; 95% CI, 1.11–1.60), and those in the soldier (OR = 1.95; 95% CI, 1.27–3.01) or housewife occupational groups (OR = 1.45; 95% CI, 1.11–1.90) tended to experience CPR practical training, whereas those who were in the 30s age group (OR = 0.71; 95% CI, 0.56–0.90), who were in the blue collar (OR = 0.73; 95% CI, 0.59–0.95), or student occupational groups (OR = 0.75; 95% CI, 0.59–0.95), and metropolitan residents (OR = 0.79; 95% CI, 0.71–0.89) were less likely to experience CPR practical training.

**Table 2:** Relationship between demographic characteristics and CPR practical training.

		CPR practical training				
	Total	Yes		Yes		
	N	N	%w	AOR	(95% CI)	β
Total	25082	19168	76.8			
Gender						
Male	16941	12959	77.0	1.17	(1.07−1.29)	0.0401**
Female	8141	6209	76.2	1.00		Ref.
Age group (years)						
20s (19–29)	7241	6060	84.3	1.72	(1.32−2.24)	0.3380***
30s (30–39)	7632	5439	70.4	0.71	(0.56−0.90)	-0.2149**
40s (40–49)	5420	4041	73.4	0.85	(0.67−1.08)	-0.1024
50s (50–59)	3704	2847	75.2	1.00	(0.79−1.27)	-0.0005
60s and order (≥60)	1085	781	74.6	1.00		Ref.
Marital status						
Single	8521	6854	81.4	0.96	(0.76−1.21)	-0.0121
Married	15393	11451	73.4	0.93	(0.76−1.13)	-0.0224
Others	1168	863	74.0	1.00		Ref.
Education level						
College or more	12137	9248	76.3	1.36	(1.12−1.65)	0.1042**
High school	10879	8439	78.1	1.33	(1.11−1.60)	0.0979**
Less than high school	2066	1481	70.2	1.00		Ref.
Income level						
Highest quantile	9678	7503	77.9	1.08	(0.90−1.30)	0.0406
Upper quantile	7677	5813	76.0	0.99	(0.82−1.19)	-0.0056
Lower quantile	5776	4363	75.6	0.94	(0.78−1.14)	-0.0304
Lowest quantile	1951	1489	77.6	1.00		Ref.
Occupation						
White collar	9430	7131	76.0	0.94	(0.76−1.16)	-0.0599
Pink collar	3617	2893	79.8	1.16	(0.93−1.45)	0.1422
Blue collar	6372	4580	71.0	0.73	(0.59−0.89)	-0.3118**
Soldier	581	519	89.0	1.95	(1.27−3.01)	0.6490**
Student	2340	1903	82.1	0.75	(0.59−0.95)	-0.2784*
Housewife	1549	1205	78.4	1.45	(1.11−1.90)	0.3600**
Unemployed	1193	937	81.6	1.00		Ref.
Residential area						
Seoul, the capital	3002	2341	78.7	0.95	(0.83−1.09)	-0.0290
Metropolitan area	5222	3893	74.6	0.79	(0.71−0.89)	-0.1249***
Urban area	8688	6590	76.9	0.91	(0.82−1.01)	-0.0515
Rural area	8170	6344	77.5	1.00		Ref.

CPR: cardiopulmonary resuscitation, AOR: adjusted odds ratio, CI: confidence interval, and β: standardized coefficient.

***p < 0.001

**p < 0.01, and

*p < 0.05.

### Mediation effect of CPR practical training

Table 3 shows the results of the mediation analysis (a three-step logistic regression analysis) to evaluate the total effect (c), the indirect effect (ab), and the direct effect (c'). The total effect on self-efficacy was significant for demographic characteristics, including male gender, 30s and 50s age groups, college or more and high school educational levels, white collar and soldier occupations, and residents of the capital and metropolitan areas (p < 0.05) (Model 1). In Model 1, the demographic characteristics for which the total effect (c) was not significant did not satisfy the first condition of the mediation analysis. These factors were excluded from the subsequent process to identify mediation type and were specified as ‘no relation’. Among the demographic characteristics for which the total effect was significant, the indirect effect was significant for males, the 30s age group, college or more and high school education, soldiers, and residents of metropolitan areas (p < 0.05) (Model 2). Finally, we confirmed that the direct effect was smaller than the total effect (i.e., |c'| < |c|) for the demographic characteristics where indirect effects were significant (Model 3), suggesting that CPR practical training mediated the pathway from demographic traits to self-efficacy in CPR performance. The effect of the metropolitan residence on self-efficacy in CPR performance was no longer significant (β = 0.0596, p ≥ 0.05) after adjusting for CPR practical training, indicating that this relationship was completely mediated by CPR practical training. Appendix 2 summarizes the path diagrams for the mediation type classified by the degree of mediation effect (e.g., no mediation, partial mediation, and complete mediation), and presents the demographic characteristics associated with each type of mediation.

**Table 3:** Mediation effect of CPR practical training on the relationship between demographic characteristics and self-efficacy in CPR performance.

	Model 1 (estimating c)		Model 2 (estimating a)		Model 3 (estimating b and c')		
	Self-efficacy in CPR performance		CPR practical training		Self-efficacy in CPR performance		Type of mediation^†^
	AOR(95% CI)	β	AOR(95% CI)	β	AOR(95% CI)	β	
Gender							
Male	2.54(2.29−2.80)	0.2312***	1.16(1.07−1.26)	0.0374**	2.56(2.31−2.84)	0.2227***	P
Female	1.00	Ref.	1.00	Ref.	1.00	Ref.	
Age group (years)							
20s (19–29)	1.01(0.76−1.36)	0.0087	1.71(1.36−2.15)	0.3366***	0.89(0.66−1.20)	-0.0686	-
30s (30–39)	0.69(0.53−0.89)	-0.2285**	0.70(0.57−0.85)	-0.2276**	0.75(0.57−0.98)	-0.1689*	P
40s (40–49)	1.05(0.81−1.35)	0.0277	0.83(0.68−1.02)	-0.1173	1.09(0.84−1.43)	0.0526	-
50s (50–59)	1.30(1.01−1.68)	0.1631*	0.96(0.79−1.18)	-0.0236	1.31(1.01−1.70)	0.1587*	N
60s and older (≥60)	1.00	Ref.	1.00	Ref.	1.00	Ref.	
Marital status							
Single	0.96(0.74−1.25)	-0.0115	0.95(0.77−1.16)	-0.0158	0.98(0.76−1.27)	-0.0052	-
Married	0.98(0.78−1.22)	-0.0071	0.95(0.79−1.13)	-0.0163	1.00(0.80−1.25)	0.00002	-
Others	1.00	Ref.	1.00	Ref.	1.00	Ref.	
Education level							
College or more	1.39(1.12−1.73)	0.1113**	1.29(1.10−1.52)	0.0867**	1.33(1.07−1.66)	0.0918*	P
High school	1.32(1.08−1.62)	0.0948**	1.26(1.08−1.47)	0.0789**	1.27(1.04−1.56)	0.0779*	P
Less than high school	1.00	Ref.	1.00	Ref.	1.00	Ref.	
Income level							
Highest quantile	1.18(0.96−1.44)	0.0824	1.11(0.95−1.30)	0.0556	1.15(0.93−1.42)	0.0672	-
Upper quantile	1.02(0.84−1.25)	0.0118	1.00(0.86−1.17)	0.0006	1.03(0.84−1.28)	0.0155	-
Lower quantile	1.07(0.88−1.30)	0.0346	0.97(0.83−1.14)	-0.0160	1.08(0.88−1.33)	0.0389	-
Lowest quantile	1.00	Ref.	1.00	Ref.	1.00	Ref.	
Occupation							
White collar	1.24(1.00−1.55)	0.2093*	0.98(0.83−1.17)	-0.0174	1.28(1.02−1.60)	0.2236*	N
Pink collar	1.06(0.84−1.34)	0.0565	1.20(0.99−1.44)	0.1745	1.03(0.81−1.30)	0.0223	-
Blue collar	1.00(0.80−1.25)	0.0008	0.76(0.64−0.91)	-0.266**	1.10(0.88−1.39)	0.0902	-
Soldier	2.98(1.68−5.29)	1.0465**	2.04(1.41−2.95)	0.6909**	2.59(1.45−4.62)	0.8694**	P
Student	1.17(0.92−1.50)	0.1539	0.81(0.66−0.98)	-0.2103*	1.26(0.98−1.63)	0.2111	-
Housewife	1.10(0.85−1.43)	0.0914	1.40(1.11−1.76)	0.3265**	1.01(0.77−1.33)	0.0111	-
Unemployed	1.00	Ref.	1.00	Ref.	1.00	Ref.	
Residential area							
Seoul, the capital	0.79(0.69−0.91)	-0.1234**	1.01(0.90−1.14)	0.0064	0.77(0.66−0.89)	-0.1349**	N
Metropolitan area	0.84(0.74−0.96)	-0.0917**	0.81(0.74−0.89)	-0.1128***	0.89(0.78−1.01)	-0.0596	C
Urban area	0.90(0.80−1.01)	-0.0561	0.94(0.85−1.02)	-0.036	0.91(0.81−1.03)	-0.0477	-
Rural area	1.00	Ref.	1.00	Ref.	1.00	Ref.	
CPR practical training							
Yes					4.06(3.70−4.44)	0.3004***	
No					1.00	Ref.	

CPR: cardiopulmonary resuscitation; AOR: adjusted odds ratio; CI: confidence interval; and β: standardized coefficients

AOR adjusted for gender, age group, marital status, education level, income level, occupation, and residential area in Models 1 and 2. AOR additionally adjusted for CPR practical training in Model 3.

***p < 0.001

**p < 0.01, and

*p < 0.05.

†Capital letters indicate the types of mediation based on the degree of effect (- = no relation; N = no mediation; P = partial mediation; C = complete mediation).

## Discussion

This study systematically unraveled the relationship of demographic characteristics and CPR practical training with self-efficacy in CPR performance using a mediation analysis. We discovered two things through the analysis: 1) CPR practical training acted as mediator in the pathway from particular demographic characteristics (e.g., gender, age, education level, occupation, and residential area) to self-efficacy in CPR performance, and 2) there was a difference in the magnitude of the mediation effect (e.g., non, partial, or complete) of CPR practical training depending on demographic characteristic.

Previous studies have consistently dealt with the impact of demographic variation on CPR training and performance [14, 31–34]. We found that the practice rate differed by gender, age group, education level, occupation, and residential area and that the demographic differences in practice rates influenced differences in self-efficacy in CPR performance. This suggests that it is necessary to control disparity in the quality of education according to the demographic group, not just to increase the practice rate but also to improve self-efficacy. High quality and consistent education, including hands-on training, should be provided to the general public.

In Korea, CPR training has been conducted mainly in the workplace, in school and in the military. As evidence of this background, the results of our study showed that males, those with higher education, and soldiers had more experience with CPR practical training, and this difference partially mediated their higher self-efficacy. Thus, those who did not belong to these groups, including females, those with less than high school education, and unemployed individuals, were less prepared for CPR practice, which may lead to low self-efficacy in CPR performance. We also confirmed that those in their 30s have lower self-efficacy, partially mediated by their lower practice rates. Pane reported that healthy younger people are actively participating in CPR education, and Swor found that younger trainees were more likely to perform bystander CPR [10, 30]. From these results, we expect that if younger people receive manikin practice on an individual basis, they will serve as skilled and competent lifesavers during local emergencies.

An interesting result from the mediation analysis was that self-efficacy in CPR performance of among those in their 50s, and among white-collar workers was not mediated by their experience of practicing CPR. This may imply that certain personal traits facilitate self-efficacy in CPR performance, even if educational effects are excluded. Furthermore, it suggests that subjective characteristics, such as a sense of social responsibility, could potentially be another mediator in the relationship between demographics and self-efficacy [13, 35]. As CPR is a practical act that is accomplished by a complex process, it is important to understand non-educational third factors as well as the educational factors that can influence the willingness to actually perform CPR.

An increasing number of studies have been performed on regional variations in CPR performance and their impact on the bystander CPR rate [28, 31, 32]. In an Arizona study, urban area residents were more likely to be trained in CPR, but did not intend to perform CPR [11]. In a Swedish study, rural area residents showed more willingness to initiate CPR than did metropolitan area residents [36]. We also found that those living in the capital and metropolitan areas showed lower self-efficacy than did urban or rural residents. Metropolitan residents’ self-efficacy in CPR performance was completely mediated by practice training. That is, the lower self-efficacy of those living in metropolitan areas was largely a result of their lack of experience in practicing with a manikin compared with those in rural areas. The capital and metropolitan areas have high population densities, and their residents are more likely to witness someone undergoing cardiac arrest, so additional efforts are needed to increase the practice rate among urban population [31].

According to Bandura's theory, self-efficacy is a cognitive factor that influences behavioral decision-making and contributes to motivation, performance, and persistence in behavior [37]. Based on this theoretical framework, several studies dealing with simulation-based education (e.g., clinical education and education in basic life support and resuscitation) have measured self-efficacy to assess the effectiveness of such training [38–40]. The results of these studies indicated that trained group showed greater self-efficacy compared with untrained groups or showed improved post-training compared to pre-training performance [38, 39]. Furthermore, some previous studies have confirmed the relationship between self-efficacy and the probability of performing CPR. Olson et al. reported that midwives with a higher level of self-efficacy were more likely to provide resuscitation in a real practice setting [40]. Ro et al. discovered that the rate of bystander CPR and the survival of OHCA patients were higher in communities with stronger self-efficacy in CPR [6]. Previous studies described above strive to obtain theoretical and empirical support for the links between self-efficacy and performance. However, unfortunately, there is still distinct lack of direct evidence for the association between self-reported ability and actual CPR performance. Such knowledge would require challenging efforts with carefully designed studies controlling for various contextual factors that might influence the actual behaviors and their outcomes.

Through this case study of Korean nationwide data, we concluded that CPR self-efficacy was not uniform even among recently educated people, because of the indirect effect of the demographic variations in the practice rate or the direct effect of the demographic characteristics. Korea is a nation where the legal basis of CPR education for adults in the general population is yet to be fully established [9]. The following two strategies are suggested to improve CPR self-efficacy for countries like Korea: maintaining consistently high quality education to reduce demographic differences in the practice rate, and providing personalized intervention with attention to demographic characteristics to enhance the effectiveness of education. The former could be a useful strategy for increasing the proportion of self-efficacy among the whole population (i.e., widespread dissemination), and the latter could be an effective strategy for strengthening individuals’ self-efficacy (i.e., efficient dissemination).

Some limitations need to be considered in this study. There may be more than one mediator in the relationship between demographic characteristics and self-efficacy in CPR performance. However, we focused on one mediator, CPR practical training, as it was expected to have the strongest mediation effect. Another important limitation is that, we measured self-efficacy as a proxy variable for competency to perform CPR. Self-efficacy is an essential cognitive step toward performing resuscitation, and that should be correlated with intention to perform CPR [5]. However, this is based on a subjective assessment, which may result in respondent bias. Self-efficacy may not be consistent with actual practice when witnessing an OHCA, and therefore high self-efficacy may not guarantee actual practice of CPR. Further investigation is needed to delineate the contribution of self-reported abilities on actual performance of CPR.

This is the first study to investigate the pathway from demographic characteristics to self-efficacy through CPR practical training as a mediator. Here, we examined the mediating effects of practical training on various demographic characteristics as predictors. Additionally, the analysis employed large-scale nationwide survey data, containing demographic and CPR-related characteristics of representative Korean adults.

## Conclusions

Self-efficacy in CPR performance for educated bystanders is determined by the combined effect of their demographic characteristics and practical training experience. This finding suggests that the gradual and systematic educational strategy centered on high quality, individualized CPR practice for the adult population should be prioritized. Providing practice-based CPR training programs optimized for trainees’ demographic characteristics will improve the effectiveness of CPR training and will ultimately contribute to increasing bystander CPR and the survival rate of patients who undergo OHCA.

## Supporting information

S1 Fig Path diagrams from demographic characteristics to self-efficacy in CPR performance via CPR practical training by mediation type. (a) No mediation; N. (b) Partial mediation; P. (c) Complete mediation; C. The solid lines indicate the significant effect and the dashed lines indicate the insignificant effect for each path. Demographic characteristics correspond to each type of mediation effect: (a) Age group (50s); occupation (white collar); residential area (the capital). (b) Gender (male); age group (30s); education level (college or more, high school); occupation (soldier). (c) Residential area (metropolitan area).
(TIF)

S1 Table Relationship between CPR training related factors and self-efficacy in CPR performance. (DOCX)
