# Supplementary figures and images for: A mediation analysis of the effect of practical training on the relationship between demographic factors, and bystanders’ self-efficacy in CPR performance
Source: PLoS One. 2019 Apr 29;14(4):e0215432. doi: 10.1371/journal.pone.0215432 (PMC6488056; doi:10.1371/journal.pone.0215432)

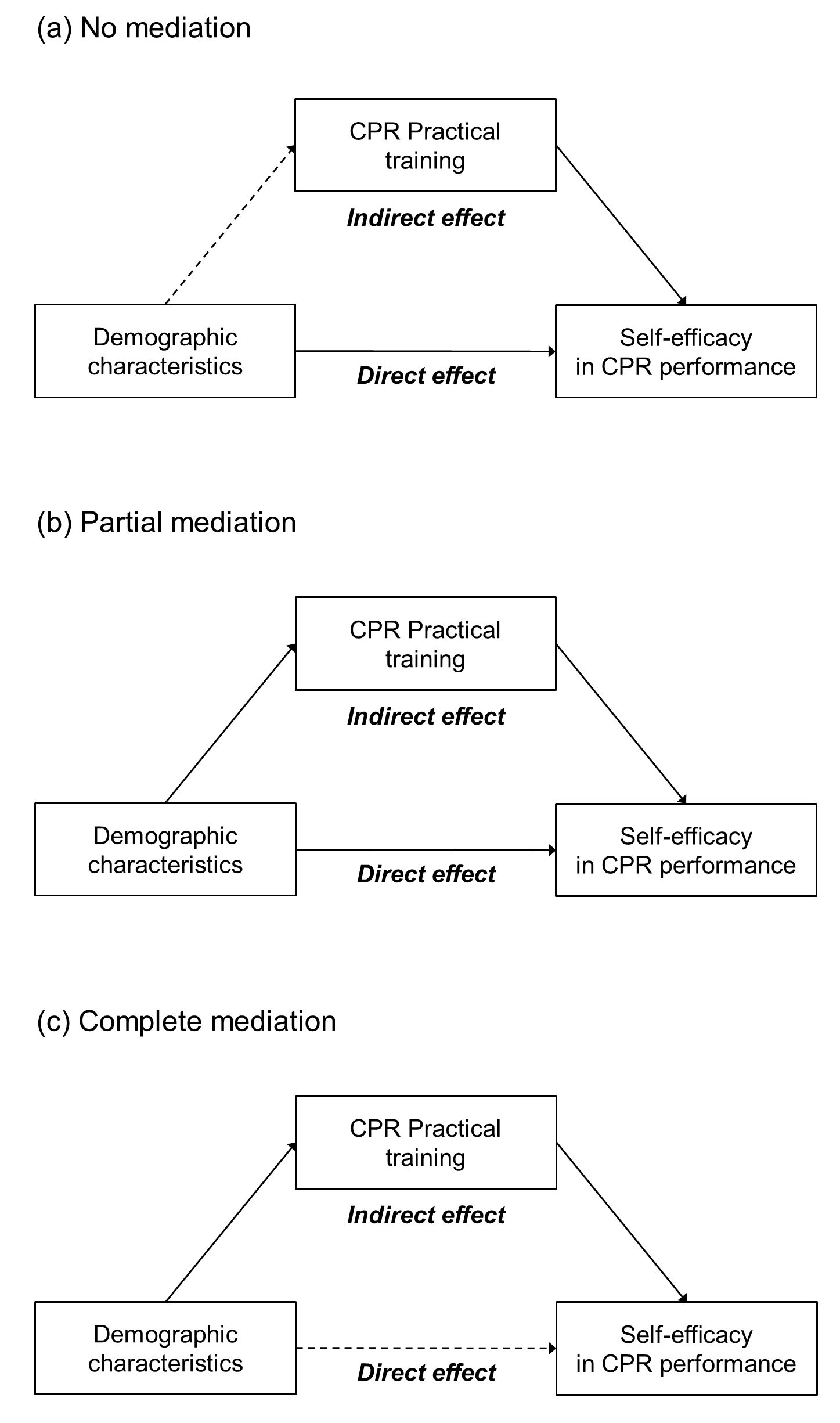

Supplement: S1 Fig — (a) No mediation; N. (b) Partial mediation; P. (c) Complete mediation; C. The solid lines indicate the significant effect and the dashed lines indicate the insignificant effect for each path. Demographic characteristics correspond to each type of mediation effect: (a) Age group (50s); occupation (white collar); residential area (the capital). (b) Gender (male); age group (30s); education level (college or more, high school); occupation (soldier). (c) Residential area (metropolitan area). (TIF) [file pone.0215432.s001.tif]
