# Supplementary material for: A mediation analysis of the effect of practical training on the relationship between demographic factors, and bystanders’ self-efficacy in CPR performance
Source: PLoS One. 2019 Apr 29;14(4):e0215432. doi: 10.1371/journal.pone.0215432 (PMC6488056; doi:10.1371/journal.pone.0215432)
Supplement: S1 Table — (DOCX) [file pone.0215432.s002.docx]

**S1 Table**. Relationship between CPR training related factors and self-efficacy in CPR performance.

|  |  | Total | Self-efficacy in CPR performance, Yes | | |
| --- | --- | --- | --- | --- | --- |
|  |  | N | N(%) | OR(95% CI) | OR(95% CI) |
| Total | | 149444 | 71504(47.8) |  |  |
| Have you trained CPR within recent 2 years? | |  |  |  |  |
|  | No | 124362 | 49501(39.8) | 1.00 |  |
|  | Yes | 25082 | 22003(87.7) | 8.61(8.26–8.97) |  |
| Were you trained CPR with manikin practice? | |  |  |  |  |
|  | No | 5914 | 4378(74.0) | 3.29(3.09−3.50) | 1.00 |
|  | Yes | 19168 | 17625(92.0) | 13.97(13.23−14.76) | 4.11(3.79−4.46) |

CPR: cardiopulmonary resuscitation, OR: crude odds ratio, and CI: confidence interval.
